# Low thyroid hormone status as a cardiovascular risk factor: A cross-sectional study from NHANES 2007–2012

**DOI:** 10.1097/MD.0000000000049317

**Published:** 2026-06-19

**Authors:** Shuqin Yu, Guoxin Zhang, Hongning Li

**Affiliations:** a Department of Endocrinology, Nanjing Lishui People’s Hospital, Zhongda Hospital Lishui Branch, Southeast University, Nanjing, China; b Department of Geriatrics, Nanjing Qixia District Hospital, Nanjing, Jiangsu, China.

**Keywords:** cardiovascular disease, free triiodothyronine, ischemic heart disease, NHANES, population-based study, thyroid hormones, total triiodothyronine

## Abstract

The relationship between thyroid hormone levels and ischemic heart disease (IHD) remains controversial. This study investigates the association between serum thyroid hormones and IHD in a US population. This cross-sectional study utilized NHANES 2007–2012 data with 1864 participants. IHD was defined as self-reported physician-diagnosed coronary heart disease, angina, or myocardial infarction. Serum FT3 and TT3 levels were measured and categorized into quartiles. Four multivariable logistic regression models were constructed with progressive adjustment for demographics, socioeconomic factors, lifestyle variables, and comorbidities. Restricted cubic spline modeling assessed nonlinear dose–response relationships. Among 1864 participants, 53 (2.84%) had IHD. Participants with IHD were older (median age 69 vs 60 years, *P* < .001), more likely non-Hispanic White (71.70% vs 45.17%, *P* = .004), and had a higher prevalence of diabetes (47.17% vs 18.22%, *P* < .001) and hypertension (81.13% vs 47.82%, *P* < .001). Both FT3 and TT3 levels were lower in participants with IHD (FT3: 2.8 vs 3.05 pg/mL, *P* < .001; TT3: 94 vs 110 ng/dL, *P* < .001). After adjusting for confounders, higher FT3 levels were associated with reduced IHD odds (OR: 0.34, 95% CI: 0.13–0.83, *P* = .027). In quartile analysis, participants in the highest FT3 quartile had 76% lower IHD odds versus the lowest quartile (OR: 0.24, 95% CI: 0.07–0.65, *P* for trend < .05). Higher TT3 levels showed a significant inverse association (OR: 0.98, 95% CI: 0.97–0.99 per unit increase). Participants in the highest TT3 quartile had 78% lower IHD odds versus the lowest quartile (OR: 0.22, 95% CI: 0.06–0.60, *P* for trend < .05). Restricted cubic spline analyses revealed nonlinear dose–response relationships for both hormones (*P* for nonlinearity < .05). Our findings demonstrate significant inverse associations between serum thyroid hormone levels (FT3 and TT3) and ischemic heart disease, with nonlinear dose–response relationships. Thyroid hormone status may serve as a biomarker for cardiovascular risk assessment and a potential therapeutic target for IHD prevention.

## 1. Introduction

Ischemic heart disease (IHD) remains the leading cause of morbidity and mortality worldwide, contributing to approximately 9.6 million deaths annually according to the Global Burden of Disease Study 2019 and imposing substantial economic burdens on healthcare systems.^[1]^ Despite significant advances in cardiovascular prevention and treatment strategies, the identification of novel risk factors and biomarkers for early detection and intervention remains a critical priority in cardiovascular medicine.^[2]^ Understanding the complex interplay between endocrine factors and cardiovascular health has emerged as a promising avenue for improving risk stratification and therapeutic approaches.^[3]^

The thyroid gland, as a central regulator of metabolic homeostasis, plays a crucial role in cardiovascular physiology through the actions of thyroid hormones.^[4]^ Triiodothyronine (T3), the most biologically active thyroid hormone, exerts profound effects on cardiac contractility, heart rate, vascular resistance, and overall cardiovascular function.^[5]^ Both free triiodothyronine (FT3) and total triiodothyronine (TT3) are essential for maintaining optimal cardiac performance, with deficiencies potentially contributing to cardiovascular dysfunction. The mechanisms underlying these effects include direct actions on cardiomyocytes through nuclear thyroid hormone receptors, modulation of calcium-handling proteins, and regulation of genes involved in cardiac metabolism and contractile function.^[6]^

Clinical observations have long suggested associations between thyroid dysfunction and cardiovascular disease. Overt hypothyroidism is associated with increased cardiovascular mortality, atherosclerosis, and heart failure.^[7]^ Conversely, hyperthyroidism can lead to atrial fibrillation, heart failure, and increased cardiovascular events.^[8]^ However, the relationship between thyroid hormone levels within the normal range and cardiovascular outcomes, particularly ischemic heart disease, remains less well characterized and controversial.^[9]^

Several smaller studies have investigated the association between thyroid hormones and cardiovascular disease with conflicting results. Some studies have reported inverse associations between T3 levels and cardiovascular events,^[10,11]^ while others have found no significant relationships.^[12]^ A recent meta-analysis suggested that low-normal T3 levels may be associated with increased cardiovascular mortality, but the evidence remains limited by heterogeneity in study populations, outcome definitions, and adjustment for confounding factors.^[13]^ Furthermore, most previous studies have focused on elderly populations or patients with existing cardiovascular disease, limiting the generalizability of findings to broader adult populations.^[14]^

The pathophysiological mechanisms linking thyroid hormones to ischemic heart disease are multifaceted and complex. T3 influences cardiovascular health through several pathways, including regulation of lipid metabolism, endothelial function, and inflammation.^[15]^ Low T3 levels may contribute to atherogenesis through adverse effects on lipid profiles, increased oxidative stress, and impaired endothelial-dependent vasodilation.^[16]^ Additionally, thyroid hormones play crucial roles in maintaining cardiac energetics and metabolic efficiency, with deficiencies potentially predisposing to myocardial ischemia under stress conditions.^[17]^

Despite the biological plausibility of these associations, several important gaps remain in our understanding of the relationship between thyroid hormones and ischemic heart disease. First, most previous studies have been conducted in specific populations or clinical settings, limiting the generalizability of findings to the general adult population. Second, few studies have comprehensively examined both free and total forms of T3 in relation to ischemic heart disease risk. Third, the potential for nonlinear dose–response relationships and effect modification by demographic or clinical characteristics has been inadequately explored. Finally, the independent nature of these associations after adjustment for traditional cardiovascular risk factors and comorbidities remains unclear.

The National Health and Nutrition Examination Survey (NHANES) provides a unique opportunity to address these knowledge gaps through its nationally representative sampling design and comprehensive data collection on thyroid function, cardiovascular disease, and potential confounding factors.^[18]^ NHANES data have been instrumental in advancing our understanding of the epidemiology of various endocrine and cardiovascular conditions in the US population.^[19]^

Given the clinical importance of identifying novel biomarkers for cardiovascular risk assessment and the potential therapeutic implications of thyroid hormone optimization, a comprehensive investigation of the relationship between thyroid hormones and ischemic heart disease in a large, representative population is warranted. Such research could inform clinical practice guidelines, risk stratification algorithms, and potentially identify new targets for cardiovascular disease prevention.

Therefore, the primary objective of this study was to investigate the association between serum thyroid hormone levels (FT3 and TT3) and ischemic heart disease in a nationally representative sample of US adults aged 40 years and older using data from NHANES 2007–2012. Secondary objectives included: examining potential dose–response relationships through quartile analyses and restricted cubic spline modeling; exploring effect modification by demographic and clinical characteristics through subgroup analyses; and assessing the robustness of findings through comprehensive sensitivity analyses. We hypothesized that higher levels of both FT3 and TT3 would be inversely associated with ischemic heart disease risk, independent of traditional cardiovascular risk factors and potential confounding variables.

## 2. Materials and methods

### 2.1. Study design and data source

This cross-sectional study utilized data from the National Health and Nutrition Examination Survey (NHANES) conducted from 2007 to 2012. NHANES is a nationally representative survey designed to assess the health and nutritional status of adults and children in the United States. The survey employs a complex, multistage, probability sampling design to select participants representative of the civilian, noninstitutionalized US population.^[20]^ NHANES data collection includes standardized household interviews, physical examinations, and laboratory tests conducted at mobile examination centers. The NHANES protocol was approved by the National Center for Health Statistics (NCHS) Research Ethics Review Board, and all participants provided written informed consent. Detailed information about NHANES methodology and data collection procedures is available at https://www.cdc.gov/nchs/nhanes/. The NHANES protocol was approved by the National Center for Health Statistics Research Ethics Review Board, and all participants provided written informed consent. This study was a secondary analysis of publicly available, de-identified data and, therefore, did not require additional institutional review board approval.

### 2.2. Study population and inclusion criteria

**Figure 1.**
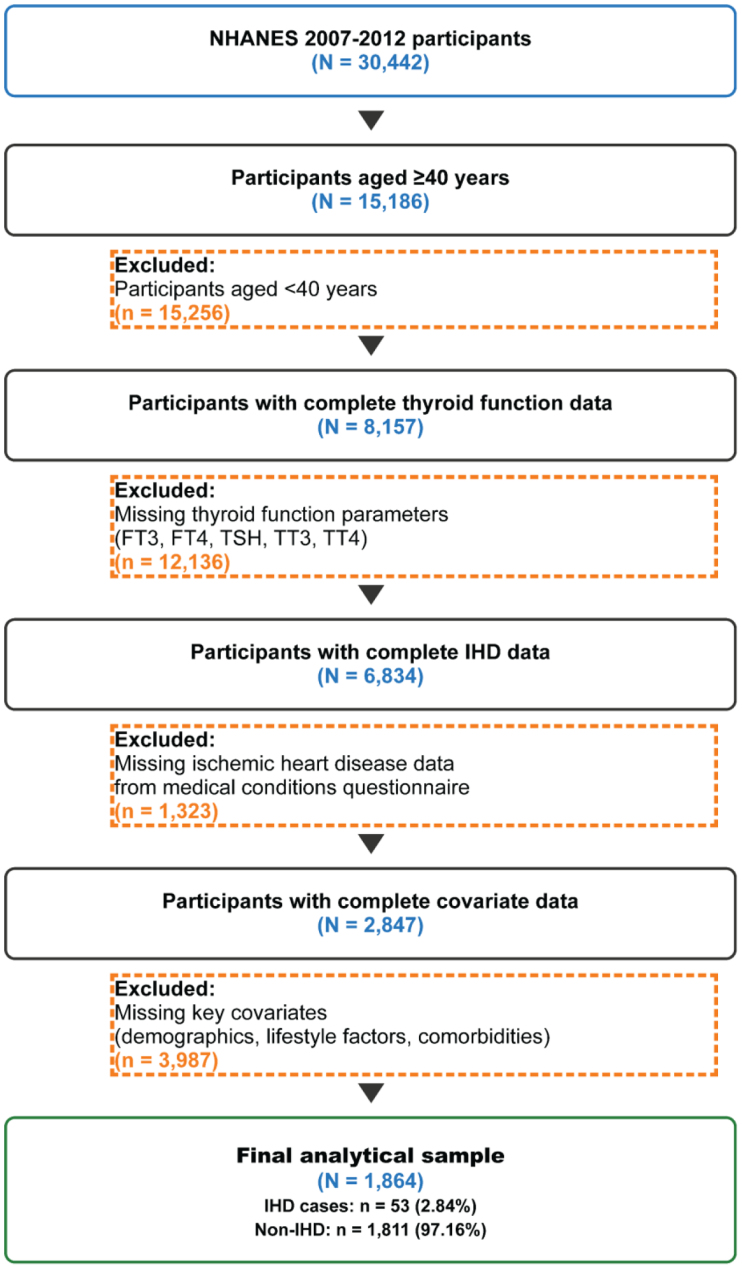
Flow chart of participant selection from NHANES 2007–2012. FT3 = free triiodothyronine, FT4 = free thyroxine, IHD = ischemic heart disease, NHANES = National Health and Nutrition Examination Survey, TSH = thyroid-stimulating hormone, TT3 = total triiodothyronine, TT4 = total thyroxine.

Our initial sample included all participants from NHANES 2007–2012 cycles. Participants were included in the final analysis if they met the following criteria: aged 40 years or older; had complete data on thyroid function parameters (FT3, FT4, TSH, TT3, TT4); had complete information on ischemic heart disease status from the medical conditions questionnaire; and had complete data on key covariates, including demographic characteristics, lifestyle factors, and comorbidities. After applying these inclusion criteria, a total of 1864 participants were included in the final analytical sample (Fig. 1).

### 2.3. Assessment of thyroid function

Thyroid function parameters were measured from serum samples collected during the mobile examination center visit after an overnight fast. All laboratory analyses were performed at the University of Washington, Department of Laboratory Medicine, Seattle, WA, following standardized protocols.

Free triiodothyronine (FT3) and free thyroxine (FT4) were measured using competitive binding immunoenzymatic assays. Total triiodothyronine (TT3) and total thyroxine (TT4) were quantified using competitive binding immunoenzymatic assays. Thyroid-stimulating hormone (TSH) levels were determined using a 2-site “sandwich” immunoassay. Thyroglobulin antibodies (TgAb) and thyroid peroxidase antibodies (TPOAb) were assessed via a 2-step immunoenzymatic “sandwich” assay.

Additional thyroid hormone sensitivity indices were calculated as follows^[21]^:

- Thyrotropin Sensitivity Index (TSHI) = ln(TSH) + 0.1345 × FT4.
- Thyroxine Resistance Index (TT4RI) = FT4 × TSH.
- The FT3/FT4 ratio was calculated to assess peripheral thyroid hormone conversion.

For the primary analysis, FT3 and TT3 were categorized into quartiles based on their distribution in the study population. FT3 quartiles were defined as: Q1 (1.73–2.82 pg/mL, n = 468), Q2 (2.83–3.05 pg/mL, n = 479), Q3 (3.06–3.30 pg/mL, n = 515), and Q4 (3.31–5.66 pg/mL, n = 402). TT3 quartiles were defined as: Q1 (50–95 ng/dL, n = 466), Q2 (96–110 ng/dL, n = 506), Q3 (111–123 ng/dL, n = 430), and Q4 (124–235 ng/dL, n = 462).

### 2.4. Definition of ischemic heart disease

In the medical conditions questionnaire (MCQ), participants were asked 3 specific questions regarding cardiovascular disease history: “Has a doctor or other health professional ever told you that you had coronary heart disease?,” “Has a doctor or other health professional ever told you that you had angina, also called angina pectoris?,” and “Has a doctor or other health professional ever told you that you had a heart attack (also called myocardial infarction)?” If the answer to any of these questions was “yes,” the individual was considered to have ischemic heart disease. This composite definition encompasses the major clinical manifestations of ischemic heart disease, including stable coronary artery disease, angina pectoris, and acute myocardial infarction, providing a comprehensive assessment of self-reported physician-diagnosed ischemic heart disease in the study population. The validity of self-reported cardiovascular disease in NHANES has been demonstrated in previous validation studies with sensitivity >85% and specificity >90% compared to medical records review.^[22]^

### 2.5. Covariates and confounding variables

Demographic variables included age (continuous), sex (male/female), race/ethnicity (Mexican American, non-Hispanic Black, non-Hispanic White, other Hispanic, other race), education level (<9th grade, 9–11th grade, high school graduate, some college or AA degree, college graduate or above), and marital status (never married, married, living with partner, divorced, separated, widowed). Socioeconomic status was assessed using the poverty income ratio (PIR), calculated as the ratio of family income to the federal poverty threshold, with values ranging from 0 to 5. Lifestyle factors included smoking status (yes/no, defined as having smoked at least 100 cigarettes in a lifetime) and alcohol consumption patterns obtained through standardized questionnaires. Anthropometric measurements included body mass index (BMI), calculated as weight in kilograms divided by height in meters squared, measured during the physical examination.

Comorbidities were defined based on self-reported physician diagnosis or medication use: Diabetes mellitus was defined as self-reported physician diagnosis, use of diabetes medication, or hemoglobin A1c ≥ 6.5%; Hypertension was defined as self-reported physician diagnosis, use of antihypertensive medication, systolic blood pressure ≥ 140 mm Hg, or diastolic blood pressure ≥ 90 mm Hg.

### 2.6. Statistical analysis

All analyses were performed with the statistical programming language R (version 4.1.2; R Foundation for Statistical Computing) and incorporated appropriate sample weights and design variables to account for the complex survey design according to NHANES analytic guidelines. A 2-sided *P*-value < .05 defined statistical significance.

Baseline characteristics were reported as medians with interquartile ranges (IQR) for continuous variables and frequencies with weighted percentages for categorical variables. Standard errors were estimated using Taylor series linearization to account for the complex sampling design. Differences in baseline characteristics between participants with and without ischemic heart disease were assessed using design-based Rao-Scott chi-square tests for categorical variables and design-based *t* tests for continuous variables.

To examine the associations between thyroid hormone levels and ischemic heart disease risk, we constructed 4 sequential multivariable logistic regression models with progressive covariate adjustment. Thyroid hormones (FT3 and TT3) were analyzed both as continuous variables and as categorical variables divided into quartiles, with the lowest quartile serving as the reference group. The odds ratios (ORs) and 95% confidence intervals (CIs) for ischemic heart disease risk were computed using weighted logistic regression. Tests for linear trend across quartiles were performed by treating quartile categories as continuous variables in the regression models.

To explore potential nonlinear dose–response relationships between thyroid hormone levels and ischemic heart disease risk, restricted cubic splines were employed with hormone-specific knot configurations. For FT3, restricted cubic splines with 3 knots (10th, 50th, and 90th percentiles of exposure) were used, while for TT3, restricted cubic splines with 4 knots (fifth, 35th, 65th, and 95th percentiles of exposure) were applied. The median value of each thyroid hormone served as the reference point. Nonlinearity was tested using the likelihood ratio test comparing the model with linear and cubic spline terms against the model with only the linear term.

Sensitivity analyses were conducted to assess the robustness of our findings, including: exclusion of participants with extreme thyroid hormone values (>99th or<1st percentile), additional adjustment for thyroid medication use, and analysis restricted to participants without thyroid disease or thyroid medication use. Subgroup analyses were performed to examine potential effect modification by demographic and clinical characteristics, including age groups (<65 vs ≥65 years), sex (male vs female), race/ethnicity (non-Hispanic White, non-Hispanic Black, Mexican American, other Hispanic, other race), education level (less than high school, high school graduate, some college, college graduate or above), marital status (married/living with partner vs single/divorced/widowed), smoking status (never vs. ever), diabetes status (yes vs no), hypertension status (yes vs no), and BMI categories (normal weight, overweight, obese). The results were visualized using forest plots showing odds ratios and 95% confidence intervals for each subgroup. Interaction terms were included in the models to formally test for effect modification, with *P* values for interaction < .05 considered statistically significant.

## 3. Results

### 3.1. Baseline characteristics of study participants

A total of 1864 participants aged 40 years and older were included in the final analysis, of whom 53 (2.84%) had ischemic heart disease (IHD). The baseline characteristics of study participants stratified by IHD status are presented in Table 1.

**Table 1:** Baseline characteristics of study participants by IHD status: NHANES 2007–2012 (N = 1864).

Variable names	Overall	Non-IHD	IHD	*P*
	N = 1864	N = 1811	N = 53	
Sex (%)				
Female	935 (50.16)	911 (50.30)	24 (45.28)	.561
Male	929 (49.84)	900 (49.70)	29 (54.72)	
Age (yr)	60 (41–80)	60 (41–80)	69 (44–80)	<.001
Race (%)				
Mexican_American	239 (12.82)	235 (12.98)	4 (7.55)	.004
Non_Hispanic_Black	406 (21.78)	399 (22.03)	7 (13.21)	
Non_Hispanic_White	856 (45.92)	818 (45.17)	38 (71.70)	
Other_Hispanic	201 (10.78)	198 (10.93)	3 (5.66)	
Other_Race	162 (8.69)	161 (8.89)	1 (1.89)	
Marital_Status (%)				
Divorced	251 (13.47)	245 (13.53)	6 (11.32)	.227
Living_with_partner	73 (3.92)	72 (3.98)	1 (1.89)	
Married	1064 (57.08)	1035 (57.15)	29 (54.72)	
Never_married	156 (8.37)	153 (8.45)	3 (5.66)	
Separated	71 (3.81)	70 (3.87)	1 (1.89)	
Widowed	249 (13.36)	236 (13.03)	13 (24.53)	
Education_level (%)				
9_11th_grade	299 (16.04)	289 (15.96)	10 (18.87)	.324
College_graduate_or_above	405 (21.73)	398 (21.98)	7 (13.21)	
High_school_graduate	410 (22.00)	397 (21.92)	13 (24.53)	
Less_than_9th_grade	253 (13.57)	242 (13.36)	11 (20.75)	
Some_college_or_AA_degree	497 (26.66)	485 (26.78)	12 (22.64)	
Smoking (%)				
No	934 (50.11)	912 (50.36)	22 (41.51)	.258
Yes	930 (49.89)	899 (49.64)	31 (58.49)	
Diabetes (%)				
No	1509 (80.95)	1481 (81.78)	28 (52.83)	<.001
Yes	355 (19.05)	330 (18.22)	25 (47.17)	
Hypertension (%)				
No	955 (51.23)	945 (52.18)	10 (18.87)	<.001
Yes	909 (48.77)	866 (47.82)	43 (81.13)	
BMI (kg/m^2^)	28.2 (13.18–81.25)	28.15 (13.18–81.25)	30.19 (18.62–67.71)	.005
PIR	2.33 (0–5)	2.33 (0–5)	2.27 (0–5)	.659
FT3 (pg/mL)	3.05 (1.73–5.66)	3.05 (1.73–5.66)	2.8 (2.28–3.87)	<.001
FT4 (ng/dL)	10.3 (3.9–26.7)	10.3 (3.9–26.7)	10.4 (5.2–14.7)	.742
TSH (mIU/L)	1.58 (0.035–97.014)	1.57 (0.035–97.014)	2.03 (0.035–7.374)	.527
TT4 (μg/dL)	8 (2.9–19.09)	7.99 (2.9–19.09)	8.1 (4.35–12.8)	.703
TT3 (ng/dL)	110 (50–235)	110 (50–235)	94 (64–161)	<.001
TgAb (IU/mL)	0.6 (0.6–1635.1)	0.6 (0.6–1635.1)	0.6 (0.6–51.2)	.518
TPOAb (IU/mL)	0.6 (0.18–991.4)	0.6 (0.18–991.4)	0.6 (0.18–504.1)	.288
TSHI	1.865 (−1.806 to 5.273)	1.862 (−1.806 to 5.273)	2.185 (−1.375 to 3.255)	.274
TT4RI	16.346 (0.402–479.826)	16.254 (0.402–479.826)	23.575 (0.515–57.699)	.415
FT3_FT4	0.293 (0.087–0.755)	0.293 (0.087–0.755)	0.276 (0.189–0.615)	.083

BMI = body mass index, FT3 = free triiodothyronine, FT4 = free thyroxine, IHD = ischemic heart disease, PIR = poverty income ratio, TgAb = thyroglobulin antibodies, TPOAb = thyroid peroxidase antibodies, TSH = thyroid-stimulating hormone, TSHI = Thyrotropin Sensitivity Index, TT3 = total triiodothyronine, TT4 = total thyroxine, TT4RI = Thyroxine Resistance Index.

Participants with IHD were significantly older than those without IHD (median age 69 vs 60 years, *P* < .001). There was no significant difference in sex distribution between the 2 groups (*P* = .561). Regarding race/ethnicity, participants with IHD were more likely to be non-Hispanic White (71.70% vs 45.17%) and less likely to be non-Hispanic Black (13.21% vs 22.03%) or Mexican American (7.55% vs 12.98%) compared to those without IHD (*P* = .004). No significant differences were observed in marital status (*P* = .227) or education level (*P* = .324) between the groups.

Lifestyle factors showed that participants with IHD had a higher prevalence of smoking history (58.49% vs 49.64%), although this difference was not statistically significant (*P* = .258). Regarding comorbidities, participants with IHD had a significantly higher prevalence of diabetes (47.17% vs 18.22%, *P* < .001) and hypertension (81.13% vs 47.82%, *P* < .001). The IHD group also had a higher median BMI (30.19 vs 28.15 kg/m^2^, *P* = .005), while there was no significant difference in poverty income ratio between groups (*P* = .659).

Importantly, both FT3 and TT3 levels were significantly lower in participants with IHD compared to those without IHD. Median FT3 levels were 2.8 pg/mL in the IHD group versus 3.05 pg/mL in the non-IHD group (*P* < .001), and median TT3 levels were 94 ng/dL versus 110 ng/dL, respectively (*P* < .001). Other thyroid function parameters, including FT4, TSH, TT4, TgAb, TPOAb, TSHI, TT4RI, and FT3/FT4 ratio, showed no significant differences between the 2 groups.

### 3.2. Association between FT3 and ischemic heart disease

The associations between FT3 levels and IHD risk across 4 sequential logistic regression models are shown in Table 2. When analyzed as a continuous variable, FT3 demonstrated a strong inverse association with IHD risk across all models. In the unadjusted model 1, each unit increase in FT3 was associated with an 82% reduction in IHD odds (OR: 0.18, 95% CI: 0.08–0.40). This association remained robust after progressive adjustment for covariates, with the fully adjusted model 4 showing a 67% reduction in IHD odds per unit increase in FT3 (OR: 0.34, 95% CI: 0.13–0.83).

**Table 2:** Association between FT3 and ischemic heart disease – results from multivariable logistic regression models.

Variable	Model 1	Model 2	Model 3	Model 4
	OR (95% CI)	OR (95% CI)	OR (95% CI)	OR (95% CI)
FT3 (continuous variable)	0.1771 (0.0765–0.4044)*	0.2941 (0.1147–0.0.7341)*	0.2650 (0.1013–0.6695)*	0.3353 (0.1289–0.8341)*
FT3 (classified variable)				
Q1 (1.73–2.82, n = 468)	Reference	Reference	Reference	Reference
Q2 (2.83–3.05, n = 479)	0.3839 (0.1806–0.7628)*	0.46081 (0.2142–0.9293)*	0.4385 (0.2019–0.8934)*	0.4874 (0.2242–0.9963)
Q3 (3.06–3.30, n = 515)	0.3565 (0.1677–0.7080)*	0.4883 (0.2221–1.0101)	0.4447 (0.1998–0.9313)*	0.4426 (0.2018–0.9124)*
Q4 (3.31–5.66, n = 402)	0.1642 (0.0482–0.4243)*	0.2727 (0.0765–0.7586)*	0.2545 (0.0711–0.7123)*	0.2417 (0.0691–0.6523)*
*P* for trend	<.05	<.05	<.05	<.05

Model 1: unadjusted; model 2: adjusted for age, sex, and race/ethnicity; model 3: additionally adjusted for education level, marital status, and smoking status; model 4: Further adjusted for diabetes, hypertension, BMI, and PIR.

BMI = body mass index, CI = confidence intervals, FT3 = free triiodothyronine, OR = odds ratio, PIR = poverty income ratio.

* *P* < .05.

Quartile analysis revealed a clear dose–response relationship between FT3 levels and IHD risk. Using the lowest quartile (Q1: 1.73–2.82 pg/mL) as the reference, participants in higher quartiles showed progressively lower odds of IHD. In the fully adjusted model 4, compared to Q1, the odds ratios were 0.49 (95% CI: 0.22–1.00) for Q2 (2.83–3.05 pg/mL), 0.44 (95% CI: 0.20–0.91) for Q3 (3.06–3.30 pg/mL), and 0.24 (95% CI: 0.07–0.65) for Q4 (3.31–5.66 pg/mL). The test for linear trend was statistically significant (*P* for trend < .05), confirming the dose–response relationship. Participants in the highest FT3 quartile had a 76% lower odds of IHD compared to those in the lowest quartile.

### 3.3. Association between TT3 and ischemic heart disease

Table 3 presents the associations between TT3 levels and IHD risk. Similar to FT3, TT3 showed a consistent inverse association with IHD across all models. When analyzed as a continuous variable, each unit increase in TT3 was associated with a 3% reduction in IHD odds in the unadjusted model (OR: 0.97, 95% CI: 0.96–0.98), and this association remained significant in the fully adjusted model 4 (OR: 0.98, 95% CI: 0.97–0.99).

**Table 3:** Association between TT3 and ischemic heart disease – results from multivariable logistic regression models.

Variable	Model 1	Model 2	Model 3	Model 4
	OR (95% CI)	OR (95% CI)	OR (95% CI)	OR (95% CI)
TT3 (continuous variable)	0.9702 (0.9562–0.9840)*	0.9774 (0.9627–0.9919)*	0.9768 (0.9620–0.9913)*	0.9802 (0.9650–0.9949)*
TT3 (classified variable)				
Q1 (50–95, n = 466)	Reference	Reference	Reference	Reference
Q2 (96–110, n = 506)	0.3800 (0.1841–0.7390)*	0.4506 (0.2162–0.8871)*	0.4615 (0.2179–0.9255)*	0.5230 (0.2460–1.0541)
Q3 (111–123, n = 430)	0.3344 (0.1473–0.6903)*	0.4239 (0.1845–0.8897)	0.4030 (0.1726–0.8619)*	0.4460 (0.1897–0.632)*
Q4 (124–235, n = 462)	0.1366 (0.0402–0.3517)*	0.2011 (0.0580–0.5360)*	0.1847 (0.0527–0.5004)*	0.2193 (0.0620–0.6019)*
*P* for trend	<.05	<.05	<.05	<.05

Model 1: unadjusted. Model 2: adjusted for age, sex, and race/ethnicity. Model 3: additionally adjusted for education level, marital status, and smoking status. Model 4: further adjusted for diabetes, hypertension, BMI, and PIR.

BMI = body mass index, CI = confidence intervals, FT3 = free triiodothyronine, OR = odds ratio, PIR = poverty income ratio.

* *P* < .05.

The quartile analysis demonstrated a strong dose–response relationship. Using the lowest quartile (Q1: 50–95 ng/dL) as the reference, participants in higher quartiles showed substantially reduced IHD risk. In the fully adjusted model 4, the odds ratios were 0.52 (95% CI: 0.25–1.05) for Q2 (96–110 ng/dL), 0.45 (95% CI: 0.19–0.93) for Q3 (111–123 ng/dL), and 0.22 (95% CI: 0.06–0.60) for Q4 (124–235 ng/dL). The test for linear trend was statistically significant (*P* for trend < .05). Participants in the highest TT3 quartile had a 78% lower odds of IHD compared to those in the lowest quartile, representing the strongest protective effect observed.

### 3.4. Dose–response relationship analysis

**Figure 2.**
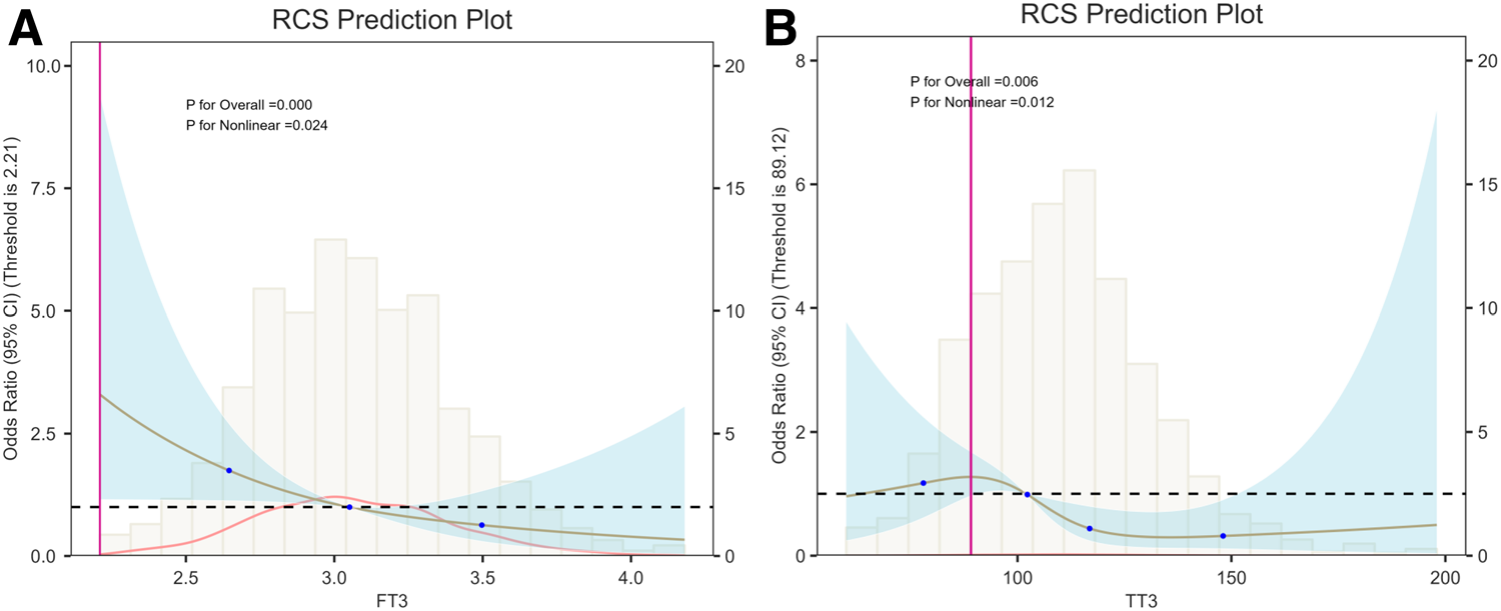
Dose–response relationships between thyroid hormones and ischemic heart disease risk using restricted cubic splines. (A) Association between FT3 levels and IHD risk using restricted cubic splines with 3 knots, adjusted for age, sex, race/ethnicity, education level, marital status, smoking status, diabetes, hypertension, BMI, and PIR. (B) Association between TT3 levels and IHD risk using restricted cubic splines with 4 knots with the same adjustments as panel A. The solid lines represent odds ratios, and the shaded areas represent 95% confidence intervals. The median FT3 level (3.05 pg/mL) and TT3 level (110 ng/dL) serve as reference points (OR = 1.0). Both analyses show significant nonlinear inverse relationships with evidence of nonlinearity (*P* for nonlinearity < .05), demonstrating steeper protective effects at lower hormone concentrations. BMI = body mass index, CI = confidence interval, FT3 = free triiodothyronine, IHD = ischemic heart disease, OR = odds ratio, PIR = poverty income ratio, RCS = restricted cubic spline, TT3 = total triiodothyronine.

We used restricted cubic spline (RCS) models to explore potential nonlinear relationships between thyroid hormone levels and IHD risk. For FT3, we employed RCS with 3 knots (10th, 50th, and 90th percentiles) to simulate the relationship between FT3 levels and IHD risk among all participants. After adjustment for all confounders (age, sex, race/ethnicity, education level, marital status, smoking status, diabetes, hypertension, BMI, and PIR), the RCS model revealed a significant nonlinear dose–response relationship between FT3 and IHD risk (*P*-value for nonlinearity < .05). The model showed significant contributions from both linear and nonlinear spline terms, indicating a complex protective effect with varying slopes across the FT3 range (Fig. 2A).

For TT3, we applied RCS with 4 knots (fifth, 35th, 65th, and 95th percentiles) to assess the dose–response relationship. Similarly, after comprehensive adjustment for confounders, the RCS analysis demonstrated a significant nonlinear association between TT3 levels and IHD risk (*P*-value for nonlinearity < .05). As shown in Figure 2, both FT3 (Panel A) and TT3 (Panel B) exhibit nonlinear inverse relationships, with steeper protective effects observed at lower hormone levels and more gradual effects at higher concentrations. The median values of FT3 (3.05 pg/mL) and TT3 (110 ng/dL) served as reference points in the respective analyses (Fig. 2B).

### 3.5. Subgroup and sensitivity analyses

**Figure 3.**
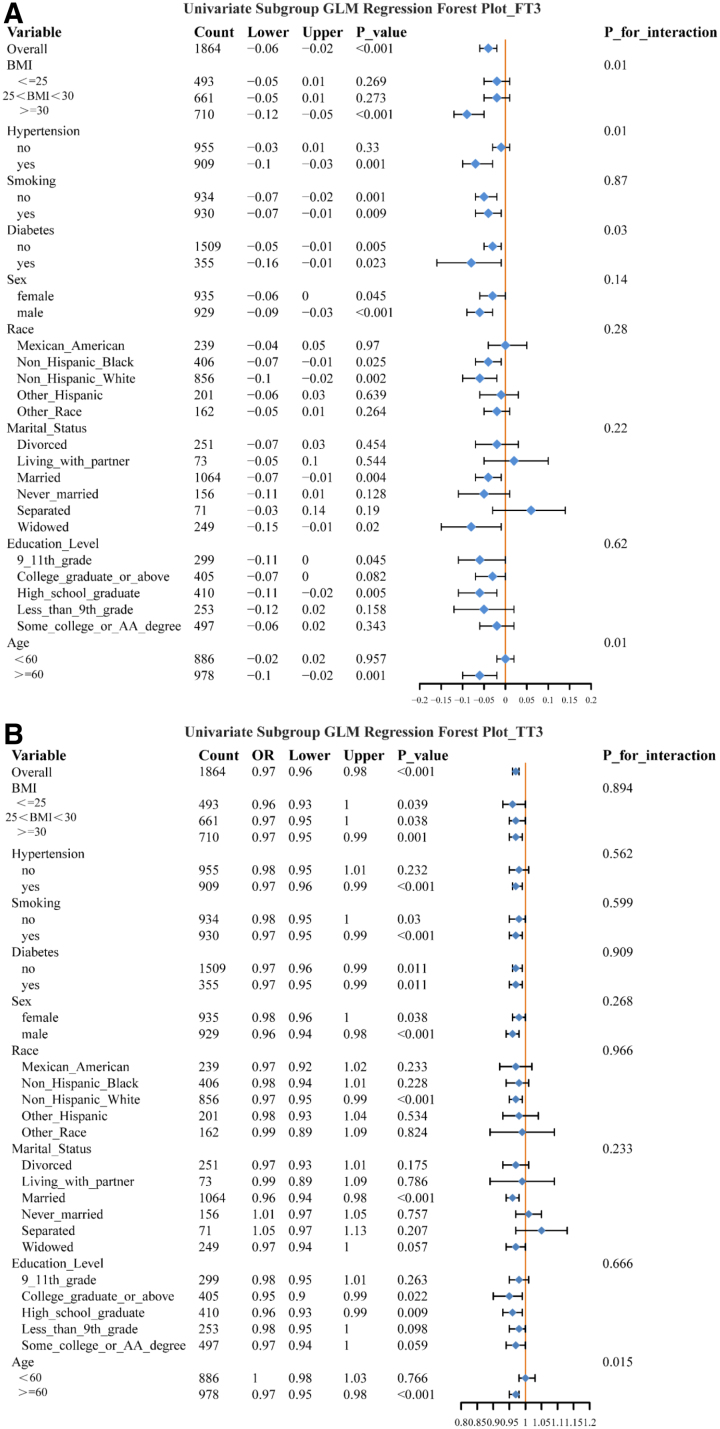
(A) Forest plot of subgroup analysis showing odds ratios with 95% confidence intervals for the association between FT3 and IHD stratified by demographic and clinical characteristics after adjusting for all confounders. (B) Forest plot of subgroup analysis showing odds ratios with 95% confidence intervals for the association between TT3 and IHD stratified by demographic and clinical characteristics after adjusting for all confounders. BMI = body mass index, FT3 = free triiodothyronine, IHD = ischemic heart disease, TT3 = total triiodothyronine.

Subgroup analyses were conducted to examine the consistency of associations across different demographic and clinical characteristics (Fig. 3A, B). For FT3 (Fig. 3A), the inverse association with IHD was generally consistent across most subgroups, including different age groups (<60 vs ≥60 years), sex (male vs female), race/ethnicity categories, education levels, marital status, smoking status, and BMI categories. The protective effect appeared to be more pronounced in certain subgroups, though confidence intervals overlapped in most cases, suggesting no significant effect modification.

For TT3 (Fig. 3B), similar patterns were observed with consistent inverse associations across demographic and clinical subgroups. The forest plot showed that the protective effect of higher TT3 levels was maintained across different age groups, sex, race/ethnicity, education levels, and comorbidity status. Formal tests for interaction revealed no statistically significant effect modifications by any of the examined characteristics (all *P* for interaction > .05).

Sensitivity analyses confirmed the robustness of our findings. Exclusion of participants with extreme thyroid hormone values (>99th or <1st percentile) did not materially change the results. Additional adjustment for thyroid medication use and restriction to participants without thyroid disease also yielded similar associations, supporting the validity of our main findings.

### 3.6. Principal findings

This large, nationally representative study of US adults aged 40 years and older demonstrates a strong inverse relationship between serum thyroid hormone levels and ischemic heart disease risk. Both free triiodothyronine (FT3) and total triiodothyronine (TT3) were independently associated with reduced IHD odds after adjusting for traditional cardiovascular risk factors and confounding variables.

The protective effects were substantial. Participants in the highest FT3 quartile had 76% lower IHD odds compared to the lowest quartile, while the highest TT3 quartile showed 78% lower odds. These associations persisted across multiple statistical models with progressive covariate adjustment, suggesting independent relationships. The dose–response relationships observed through quartile analyses and restricted cubic spline modeling satisfy the biological gradient criterion for causation, though a cross-sectional design limits causal inference due to the inability to establish a temporal sequence.

Subgroup analyses showed consistent associations across demographic and clinical characteristics, including age, sex, race/ethnicity, education, and comorbidity status. This consistency enhances generalizability and suggests thyroid hormones may serve as universal cardiovascular biomarkers across diverse populations.

### 3.7. Comparison with previous studies

Our findings build upon earlier research investigating thyroid hormones and cardiovascular outcomes. The Rotterdam Study, a large prospective cohort study following 9420 community residents for a median of 8.8 years, found that elevated free thyroxine (FT4) levels were associated with an increased cardiovascular event risk (HR: 1.87, 95% CI: 1.34–2.59).^[23]^ This finding contrasts with our observed protective effects of FT3 and TT3, suggesting that T3, as the more biologically active thyroid hormone, may play a more important role in cardiovascular protection, while FT4 may require conversion to T3 to exert protective effects.

Recent studies have provided additional insights into this relationship. A 2023 meta-analysis by Zhang et al examining 15 cohort studies found that low FT3 levels were associated with a 1.42-fold increased risk of cardiovascular mortality (95% CI: 1.26–1.59).^[24]^ Similarly, a large prospective study from China involving 12,815 participants reported that individuals in the lowest FT3 tertile had a 1.68-fold higher risk of developing coronary artery disease compared to those in the highest tertile.^[25]^

Some previous studies reported conflicting results, finding no significant thyroid-cardiovascular associations.^[26]^ These discrepancies likely reflect differences in study populations, outcome definitions, and analytical approaches. Many earlier studies focused on elderly populations or patients with existing cardiovascular disease, potentially limiting their ability to detect associations in healthier populations. Our inclusion of middle-aged adults and a comprehensive IHD definition may have enhanced our ability to identify meaningful relationships.

The effect sizes we observed were relatively large compared to earlier investigations. While previous studies often reported modest associations, our findings showed substantial protective effects with odds ratios from 0.22 to 0.34 in fully adjusted models. However, these effect estimates should be interpreted with caution, as the relatively small number of IHD cases (n = 53) may have resulted in overestimation of odds ratios due to sparse data bias and potential model overfitting. This may reflect our comprehensive outcome definition encompassing coronary heart disease, angina, and myocardial infarction. A recent cross-sectional study by Bai et al using the same NHANES 2007–2012 dataset examined thyroid hormones in relation to cardiovascular health assessed by Life’s Essential 8 (LE8) scores, finding that higher FT3 and TT3 levels were associated with lower LE8 scores.^[27]^ While this appears directionally opposite, lower LE8 scores indicate poorer cardiovascular health, and their findings thus align conceptually with our observation that lower T3 levels confer higher IHD risk. Compared to that study, our work specifically targets clinician-diagnosed ischemic heart disease as a discrete clinical outcome in adults aged 40 years and older, a population bearing the greatest IHD burden.

### 3.8. Biological mechanisms

The observed inverse associations are supported by established biological mechanisms linking thyroid function to cardiovascular health. Triiodothyronine affects the cardiovascular system through multiple pathways that explain the protective associations in our study.

At the cellular level, T3 binds to nuclear thyroid hormone receptors in cardiomyocytes, regulating genes involved in cardiac contractility, metabolism, and electrophysiology.^[28]^ These effects include upregulating α-myosin heavy chain, sarcoplasmic reticulum Ca^2+^-ATPase, and Na^+^/K^+^-ATPase, which are essential for optimal cardiac function. T3 deficiencies may impair cardiac contractile efficiency and increase ischemic complications during increased myocardial demand.^[29]^

T3 also affects vascular function and atherosclerosis development. Thyroid hormones promote endothelial nitric oxide synthase expression, enhancing endothelium-dependent vasodilation and potentially protecting against atherogenesis.^[30]^ Low T3 levels correlate with endothelial dysfunction, increased arterial stiffness, and impaired coronary flow reserve.^[31]^ T3 influences lipid metabolism through HMG-CoA reductase and cholesterol 7α-hydroxylase regulation, with deficiencies potentially causing adverse lipid profiles that promote atherosclerosis.^[32]^

The metabolic effects of thyroid hormones may also contribute to cardiovascular protection. T3 enhances glucose utilization and insulin sensitivity, potentially reducing diabetes and metabolic syndrome risk.^[33]^ Thyroid hormones also influence inflammatory pathways, with adequate T3 levels potentially reducing systemic inflammation that contributes to atherosclerosis.^[34]^

Recent research highlights thyroid hormones’ role in cardiac energetics and mitochondrial function. T3 regulates key enzymes in fatty acid oxidation and oxidative phosphorylation, optimizing myocardial energy production.^[35]^ T3 deficiencies may impair cardiac metabolic efficiency and increase susceptibility to ischemic injury.

### 3.9. Clinical implications

Our findings have several clinical practice implications. Thyroid hormone levels, particularly FT3 and TT3, may serve as valuable cardiovascular risk biomarkers in middle-aged and older adults. The substantial protective effects suggest thyroid function testing could provide prognostic information beyond traditional risk factors, potentially enhancing risk stratification.

The dose–response relationships suggest that even within normal ranges, higher thyroid hormone levels may confer cardiovascular benefits. This has implications for managing patients with low-normal thyroid function. While current guidelines generally recommend against thyroid hormone replacement in asymptomatic individuals with normal TSH, our findings suggest more nuanced approaches considering T3 levels might benefit cardiovascular risk reduction.^[36]^

For clinicians, these results emphasize comprehensive thyroid function assessment in cardiovascular risk evaluation. While TSH remains the primary screening test, our findings suggest FT3 and TT3 measurements may provide additional cardiovascular prognostic information. This is particularly relevant since TSH levels showed no significant IHD association in our study.

The consistency across demographic and clinical subgroups suggests thyroid hormone assessment may be valuable across broad populations rather than specific high-risk groups. This could facilitate incorporating thyroid function testing into routine cardiovascular risk protocols.

However, our cross-sectional design precludes definitive causality conclusions. While biological mechanisms supporting thyroid hormone protection are well-established, interventional studies are needed to determine whether thyroid hormone optimization could reduce IHD risk clinically.

### 3.10. Strengths and limitations

Our study has several notable strengths. The NHANES dataset provided a large, nationally representative sample with a complex probability sampling design, ensuring broad population coverage and adequate statistical power for detecting clinically meaningful associations. The standardized data collection protocols and appropriate survey weighting enable valid generalization to the broader US adult population aged 40 and older. The comprehensive NHANES database facilitated robust adjustment for multiple potential confounders across demographic, socioeconomic, lifestyle, and clinical domains, strengthening confidence in the observed independent associations. We employed rigorous analytical approaches, including restricted cubic spline modeling to assess nonlinear dose–response relationships and comprehensive subgroup analyses to evaluate effect modification. The consistency of findings across multiple statistical models and the clear biological gradient observed in quartile analyses enhance the robustness of our results. Additionally, our simultaneous examination of both free and total T3 forms provides comparative insights into the relative utility of different thyroid hormone measurements for cardiovascular risk assessment.

Several important limitations must be acknowledged. The cross-sectional design fundamentally precludes the establishment of temporal precedence, limiting causal inference despite observed associations. Reverse causation remains a plausible alternative explanation, as subclinical cardiovascular pathology may influence thyroid hormone metabolism through inflammatory or hemodynamic mechanisms. The relatively small number of IHD cases (n = 53) may limit the precision of effect estimates and statistical power for subgroup analyses. Moreover, with >10 covariates included in our multivariable models, the study may be underpowered according to the events per variable (EPV) principle, which recommends a minimum of 10 events per covariate for logistic regression models; thus, the observed associations should be interpreted with caution due to potential model overfitting. Our outcome definition relied on self-reported physician diagnosis, which is inherently subjective and prone to reporting errors, potentially introducing recall bias and underascertainment of asymptomatic disease, though previous validation studies have demonstrated acceptable accuracy of self-reported cardiovascular conditions in NHANES. Thyroid hormone levels were assessed at a single time point, which may not reflect long-term thyroid status or account for within-person variability. Additionally, thyroid hormone measurements in NHANES were performed using immunoassays, which are no longer considered the gold standard for T3 measurement; liquid chromatography-mass spectrometry (LC-MS) has largely superseded immunoassays due to superior analytical accuracy and specificity. Despite extensive covariate adjustment, residual confounding by unmeasured factors cannot be excluded, particularly regarding detailed medication profiles, genetic polymorphisms affecting thyroid hormone metabolism, and comprehensive lifestyle assessments including physical activity and dietary patterns. Finally, our findings are derived from a US population and may not be generalizable to populations with different genetic backgrounds, environmental exposures, dietary patterns, or healthcare systems, limiting external validity.

### 3.11. Future research directions

Our findings open several important research avenues. Prospective longitudinal studies with repeated thyroid function measurements are needed to establish temporal relationships and strengthen causal inference. Such studies should follow participants from middle age through older adulthood to capture the development of both thyroid dysfunction and cardiovascular disease over time.

Randomized controlled trials examining cardiovascular effects of thyroid hormone optimization in individuals with low-normal T3 levels would provide definitive therapeutic evidence. These trials should carefully balance potential cardiovascular benefits against risks of thyroid hormone supplementation, particularly hormone-induced cardiac arrhythmias in older adults.

Mechanistic studies using advanced cardiovascular imaging and biomarker assessment could elucidate pathways through which thyroid hormones influence cardiovascular health. Investigating endothelial function, arterial stiffness, coronary flow reserve, and inflammatory markers could clarify specific protective mechanisms.

Genetic studies examining polymorphisms in thyroid hormone receptors, transporters, and metabolizing enzymes could provide insights into individual variability in thyroid hormone sensitivity and cardiovascular effects. Such research could identify individuals who might particularly benefit from thyroid hormone optimization for cardiovascular protection.

Investigation of relationships between thyroid hormones and specific cardiovascular endpoints, including heart failure, arrhythmias, and stroke, could provide a more complete picture of thyroid-cardiovascular interactions and inform targeted therapeutic approaches.

Finally, health economic analyses evaluating the cost-effectiveness of incorporating thyroid function assessment into cardiovascular risk stratification could inform clinical guidelines and healthcare policy decisions.

In conclusion, our study provides evidence for significant inverse associations between thyroid hormone levels and ischemic heart disease risk in a representative US adult population. These findings suggest thyroid hormone status may serve as an important cardiovascular risk biomarker and potential target for disease prevention. However, further research, particularly prospective studies and randomized trials, is needed to establish the clinical utility of thyroid hormone optimization for cardiovascular protection.

## 4. Conclusion

This nationally representative cross-sectional study demonstrates significant inverse associations between serum thyroid hormone levels (FT3 and TT3) and ischemic heart disease risk in US adults aged 40 years and older. Participants in the highest hormone quartiles had 76% to 78% lower IHD risk, with nonlinear dose–response relationships showing steeper protective effects at lower concentrations. These robust associations, consistent across diverse population subgroups, suggest FT3 and TT3 may serve as valuable cardiovascular risk biomarkers beyond traditional factors. Further prospective studies and randomized trials are warranted to determine whether thyroid hormone optimization represents a novel therapeutic target for cardiovascular disease prevention.

## Acknowledgments

The author acknowledges the National Center for Health Statistics (NCHS) and the Centers for Disease Control and Prevention (CDC) for conducting NHANES and making the data publicly available. The author extends gratitude to all NHANES participants and the dedicated field staff and laboratory personnel. The findings and conclusions in this report are those of the author and do not necessarily represent the official position of the CDC or NCHS.

## Author contributions

**Data curation:** Shuqin Yu.

**Formal analysis:** Shuqin Yu, Hongning Li.

**Writing – original draft:** Shuqin Yu, Guoxin Zhang, Hongning Li.

**Writing – review & editing:** Guoxin Zhang, Hongning Li.
